# A Mysterious Case of Autoimmune Hepatitis Triggered by Herpes Zoster Virus Versus Drug-Induced Liver Injury

**DOI:** 10.7759/cureus.100667

**Published:** 2026-01-03

**Authors:** Samia Alajab, Sara Tariq, Osamede Agho, Lela Adeoshun, Lizeth Diaz Ledesma

**Affiliations:** 1 Internal Medicine, Guthrie Lourdes Hospital, Binghamton, USA; 2 Internal Medicine, Igbinedion University Medical School, Benin, NGA

**Keywords:** cholestatic liver injury, drug induced liver injur, hepatitis autoimmune, herpes zoster virus, liver injury

## Abstract

Autoimmune hepatitis triggered by Herpes Zoster infection is a relatively rare condition and a diagnosis of exclusion, as there are other more common causes of autoimmune hepatitis. While evaluating a patient with suspected autoimmune hepatitis, common causes such as viral hepatitis, Epstein-Barr virus, and medications like amoxicillin-clavulanic acid, statins, nitrofurantoin and methyldopa must be ruled out. Furthermore, autoimmune processes like thyroiditis, type 1 diabetes mellitus, rheumatoid arthritis, and ulcerative colitis should be considered. Herpes Zoster virus is known to cause diseases such as post-herpetic neuralgia, meningitis, meningoencephalitis, myelitis, cranial neuropathies, keratitis, uveitis, scleritis, and vision loss. Rarely, it can also trigger an autoimmune process in the body, causing hepatitis. A major dilemma that can be encountered while evaluating a patient with suspected autoimmune hepatitis precipitated by Herpes Zoster infection is drug-induced liver injury, especially if the patient had been managed with a medication known to cause liver injury. Both drug-induced liver injury and Herpes Zoster hepatitis have overlapping clinical, laboratory and histological pictures that can pose a significant diagnostic challenge. We report a case of autoimmune hepatitis in an elderly female precipitated by Herpes Zoster infection. She was observed with findings typical of cholestatic liver disease, and an extensive workup, including imaging and liver biopsy, was done to determine the cause of liver injury. Her clinical presentation, laboratory and Imaging findings favored Herpes Zoster virus as the likely culprit for autoimmune hepatitis.

## Introduction

Autoimmune hepatitis is an inflammatory disease of the liver occurring due to abnormal immune response targeting liver antigens, progressing to liver cirrhosis and end-stage liver failure, and is more prevalent in women [1].

It can occur due to various causative agents, including drugs and viruses like herpes simplex, hepatitis, measles, and Epstein-Barr virus. Rarely, Varicella Zoster Virus (VZV) has been reported as a culprit for causing autoimmune hepatitis in genetically susceptible individuals [2].

Varicella-zoster virus is a herpes virus that causes chickenpox (varicella) and shingles (herpes zoster) and is transmitted through airborne particles and contact [3]. Drug-induced liver injury is also a rare adverse event resulting in liver damage. Some novel agents can also affect the immune response, leading to autoimmune liver injury diagnosed on serologic tests and liver biopsies [4].

We report a case of autoimmune hepatitis triggered by HZV, possibly due to cross-reactivity of viral protein with autoantigens acting against liver tissue. This molecular mimicry can damage liver parenchyma and can create a pattern similar to drug-induced liver injury on liver biopsy.

## Case presentation

An 83-year-old female with medical history significant for hypertension, osteoarthritis, syndrome of inappropriate anti-diuretic hormone (SIADH), restless leg syndrome and anxiety, presented to the emergency department (ED) with complaints of worsening neck pain and swelling. Over the past few days, she had noticed worsening neck pain, swelling, sore throat, difficulty swallowing, dry cough, and voice change. She denied fever, chills, shortness of breath, trismus, or a history of dental infection. She also complained of mild nausea without vomiting, abdominal pain, diarrhea, or constipation. She reported feeling weak in general.

Patient is a former smoker, quit 26 years ago, with no alcohol or illicit drug use. She also had a COVID infection a month prior to this presentation, which was managed with supportive measures only.

List of home medications included amlodipine 5 mg oral daily, hydrochlorothiazide 25 mg oral daily, alprazolam 0.25 mg at bedtime, vitamin D, vitamin B12 supplement, and ropinirole 0.25 mg. She was not taking any herbal supplements or over-the-counter medication apart from psyllium.

In the ED, patient vitals were stable, temperature 98.2°F, blood pressure (BP) 133/83, heart rate (HR) 69, respiratory rate (RR) 16, oxygen saturation 99% on room air. Physical examination was unremarkable except for dry mucous membranes, posterior oropharyngeal erythema without exudate, and submandibular swelling.

Initial workup showed a normal white blood cell (WBC) count. Comprehensive metabolic profile (CMP) was significant for hyponatremia, sodium 124mmol/L, (reference range 136-145 mmol/L) (patient has chronic hyponatremia at baseline), elevated liver function tests (LFTs), aspartate aminotransferase (AST) 88 U/L (reference range < 32 U/L), alanine aminotransferase (ALT) 106 U/L (reference range 5-33 U/L), alkaline phosphatase (ALP) 782 U/L (reference range 35-104 U/L), total bilirubin 4.20 mg/dl (reference range 0.00-1.10 mg/dl). Renal function was normal. TSH was 0.81 (reference range 0.27-4.20). The acetaminophen level was within normal limits. Tests for COVID-19, respiratory syncytial virus (RSV), influenza, and infectious mononucleosis (Monospot test) were negative. The rapid strep throat test was unremarkable, and the throat culture revealed normal respiratory flora. Hepatitis panel, including Hepatitis A, B, and C serologies, came back negative. The right upper quadrant abdominal ultrasound was unremarkable for any acute pathology.

She received intravenous (IV) fluids, IV antibiotic (ceftriaxone 1000 mg) and IV steroid (dexamethasone 10 mg) in the ED with significant improvement in neck swelling and sore throat. However, liver enzymes continue to trend up. By day three, the patient developed clinically concerning jaundice with scleral and skin involvement.

Further workup revealed elevated lipase 895U/L (reference range 13-60 U/L) and amylase 504 U/L (reference range 28-100 U/L). At this point, ALP was 1002 U/L, AST 270 U/L, ALT 206 U/L, gamma glutamyl transferase (GGT) 842 U/L (reference range 5-36 U/L), total bilirubin 4.6 mg/dl, with direct bilirubin 3.9 mg/dl (reference range 0.00-0.3 mg/dl). A gastroenterology consult was initiated. The patient denied any abdominal symptoms; however, she has noticed a poor appetite.

Gastroenterology suggested that there is a possibility of viral effect, although this would be more hepatocellular injury pattern. And there is no clear evidence of choledocholithiasis or cholelithiasis on imaging. However, there is elevated lipase, which may suggest the possibility of an obstructive process in the common bile duct. Based on this, magnetic resonance cholangiopancreatography (MRCP) was recommended, in addition to trending LFT, international normalized ratio (INR) and CBC daily. Autoimmune workup was also initiated with anti-mitochondrial antibodies (AMA), anti-nuclear antibodies (ANA), and anti-smooth muscle antibodies (ASMA), along with serology for Epstein-Barr Virus (EBV) and Cytomegalovirus (CMV).

Intravenous antibiotic was discontinued, and she was switched to oral amoxicillin/clavulanate (Augmentin). MRCP results did not show any obstructive pathology in the hepatobiliary region (no evidence of sclerosing cholangitis).

CMV serology testing (IgM and IgG) was negative, EBV IgG antibody titer was positive, and IgM was within normal limits. ANA, AMA, and perinuclear Antineutrophil Cytoplasmic Antibodies (p-ANCA) were negative. Anti-smooth muscle antibodies were positive; however, they could be falsely positive. The titer (1-20) is usually insignificant and non-diagnostic for autoimmune hepatitis.

By admission day 6, her liver function trended up with rising GGT at 1405, worsening jaundice with total bilirubin at 12.5. But aminotransferase levels started to decrease in the face of elevated total bilirubin and ALP. The patient is jaundiced, INR is an elevated, with no sign of hepatic encephalopathy. Significant labs are displayed in Table 1.

**Table 1:** Significant labs AST: Aspartate aminotransferase; ALT: Alanine aminotransferase; ALP: Alkaline phosphatase; GGT: Gamma-glutamyl transferase.

Significant Labs	Results On admission	Peak Results	Reference Range
Lymphocyte Count	11.0	9.1	19.3-51.7%
AST	88	398	<=30 U/L
ALT	106	370	5-33 U/L
ALP	782	1232	35-104 U/L
GGT	842	1405	5-36 U/L
Total Bilirubin	4.20	19.10	0.00-1.10 mg/dl
Direct Bilirubin	3.9	12.4	0.0-0.3 mg/dl

At this point, liver biopsy was considered to rule out infiltrative disease, such as sarcoidosis, amyloidosis, lymphoma, and, less likely, infiltrated tuberculosis. Gastroenterology also suggests assessing the patient for herpes simplex and herpes zoster virus infections in addition to triple phase computed tomography (CT) abdomen to rule out infiltrative disease.

**Figure 1:**
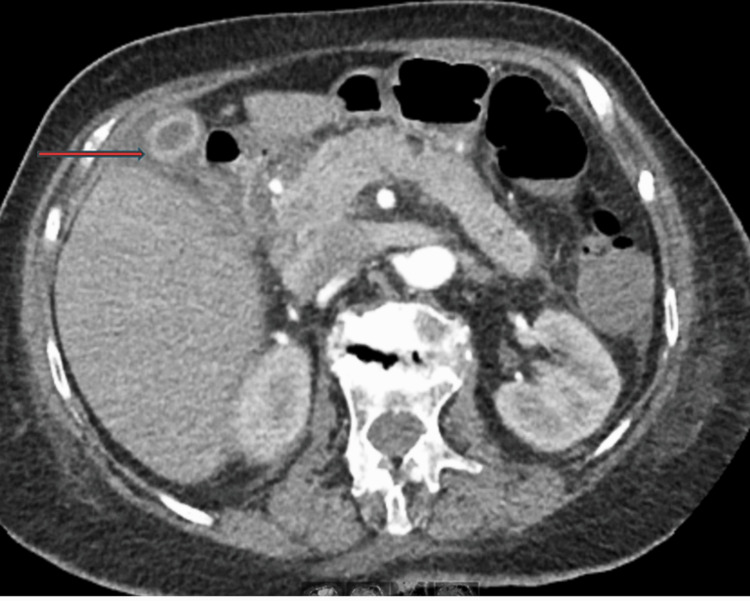
CT abdomen/pelvis with IV contrast (axial view) Prominent mural thickening/edema, measuring up to 1 cm in thickness circumferentially. Mild mucosal enhancement is seen as well as pericholecystic fluid, consistent with cholecystitis:  Red arrow

**Figure 2:**
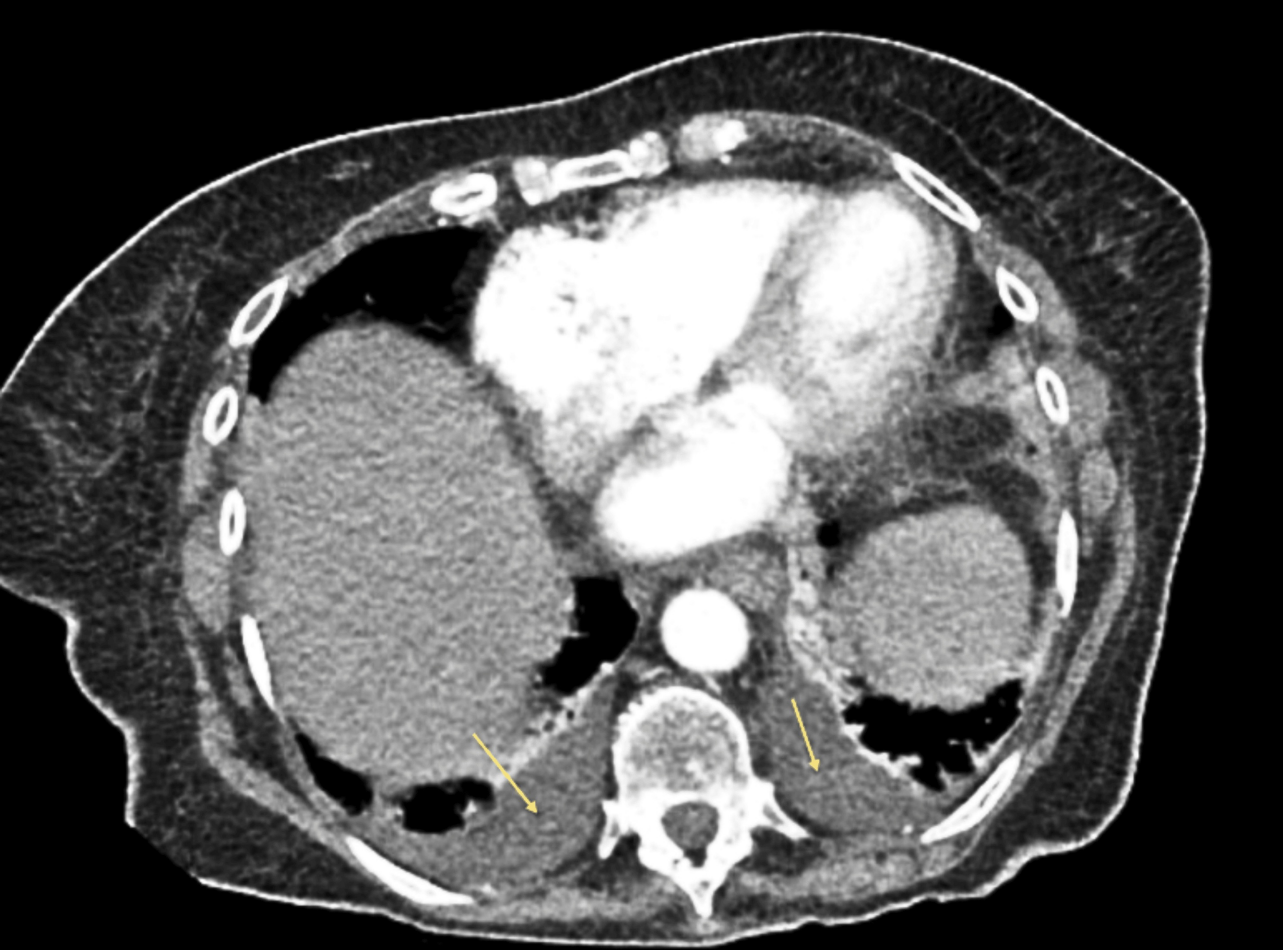
CT abdomen/pelvis with IV contrast (Axial view) Mild-moderate bilateral pleural effusions, yellow arrows

CT abdomen findings were suggestive of cholecystitis and bilateral pleural effusion with no evidence of intra- or extrahepatic malignancy, which are shown in Figures 1, 2.

Based on the CT results, she was started on IV ceftriaxone and metronidazole; however, they were discontinued after the initial dose as the patient had no clinical signs or symptoms suggestive of cholecystitis.

**Figure 3:**
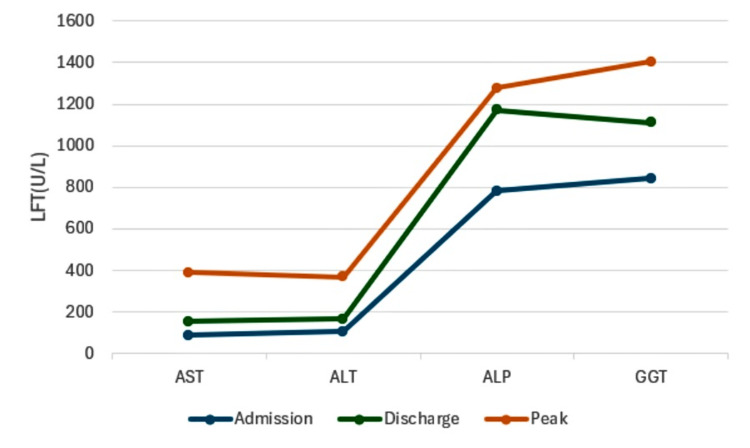
LFT markers trend LFT: Liver function test; AST: Aspartate aminotransferase; ALT: Alanine aminotransferase; ALP: Alkaline phosphatase; GGT: Gamma-glutamyl transferase; U/L: Unite Per liter

**Figure 4:**
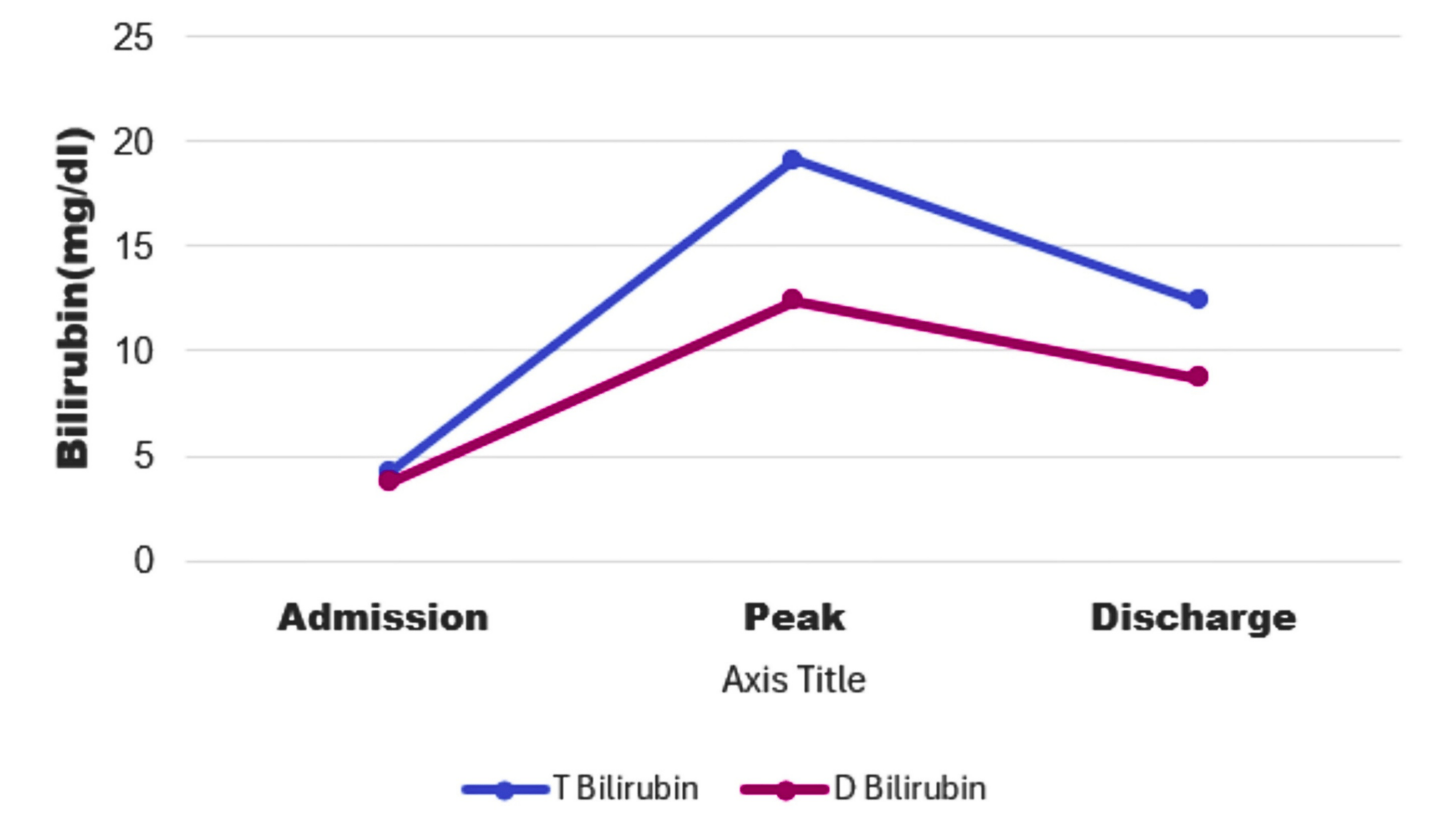
Bilirubin trend T Bilirubin: Total Bilirubin; D Bilirubin: Direct Bilirubin; MG/DL Milligram per Deciliter

Liver biopsy results revealed scattered groups of hepatocytes with cytoplasmic bile granules, indicating impaired bile excretion into colonic villi. Bile ducts were present in the portal tracts with chronic dry triaditis (inflammation of the liver, pancreas, and small intestine). The liver biopsy sample was sent to a tertiary center for a second opinion with more extensive histology as recommended by gastroenterology. At this time, total bilirubin was 16.90 mg/dl, ALP at 960 U/L, AST 177 U/L and ALT 124 U/L. LFT markers trend showed in Figures 3, 4.

Herpes simplex antibody results came back negative; however, she tested positive for varicella-zoster IgM antibody. Patient reported that she never received the zoster vaccine, and has no history of exposure. An infectious disease specialist was consulted, and she was started on acyclovir 800 mg five times daily for seven days, with repeat varicella-zoster IgM/IgG antibody within one week.

Within the next couple of days, liver function started to come down, and bilirubin improved from a peak of 19 to 11. The infectious disease specialist recommended continuing acyclovir for a total of 12-14 days. Patient remained stable, liver function continues to improve, and she was discharged on oral valacyclovir 1g three times daily to complete the total course of 14 days (IV acyclovir plus oral valacyclovir)

After discharge, liver biopsy reports from Mount Sinai revealed finding of cholestatic liver disease with damage to the bile duct, consistent with drug-induced liver injury (DILI), which further sparked concerns for an outpatient or hospital-administered drug-induced liver injury.

The detailed liver needle biopsy report showed centrilobular hepatocellular cholestasis with minimal hepatocyte injury and inflammation. Portal tracts showed lymphoplasmacytic inflammation with eosinophils, endothelitis and bile duct damage as well as lymphocytic cholangitis. Florid duct lesions, interface hepatitis, and ductular reaction are not present. Trichrome stain shows no fibrosis; iron stain is negative. PAS-D reaction is negative for cytoplasmic globules.

After an extensive review of her medication list and discussion with the patient, the only drug with the possibility of cholestatic liver implications could be amoxicillin/clavulanate, which was started after admission for her neck swelling. However, her condition improved with the administration of acyclovir, suggesting herpes zoster etiology, and despite discontinuation of amoxiclav, LFTs continued to worsen. Furthermore, the presence of anti-smooth muscle antibodies, transaminitis, and decreased biliary excretion on liver biopsy supports the diagnosis of autoimmune hepatitis. There’s also a possibility of post-COVID-19 reactivation of herpes zoster infection.

In addition, herpes zoster liver injury can mimic DILI clinically as well as on a liver biopsy with overlapping histologic features, especially if disseminated herpes zoster causes liver injury without any characteristic skin rash, which can make it a diagnostic challenge.

## Discussion

Autoimmune hepatitis has multiple triggering factors, including but not limited to drugs and viruses. The role of viruses in causing autoimmune hepatitis correlates with a cross-interaction between viral proteins and liver autoantigens [2]. Herpes Zoster Viral infection occurs as a result of reactivation of varicella zoster virus in an immunocompromised state, manifesting usually as painful vesicles in a dermatomal distribution, termed shingles [5].

A case report published by Waleed K Al-Hamoudi in 2009 showed the development of severe autoimmune hepatitis and fulminant hepatic failure in a 23-year-old man after a varicella zoster infection due to molecular mimicry between viral and liver proteins [2].

A systematic scope review by Czech T & Nishimura Y from 2022 studied 19 articles to evaluate the characteristics of herpes zoster infection in patients with COVID-19. They observed primary varicella zoster infections or reactivations in 25 patients, with 48% developing disseminated VZV infection. Patients developed primary VZV infection or reactivation, and COVID-19 was found to have low lymphocyte counts. They concluded that patients with COVID-19 are more susceptible to developing disseminated VZV infection [6].

Brewer, E. C., & Hunter, L. reported a case in 2018 of a 66-year-old female who developed acute liver failure due to varicella-zoster virus infection, due to reactivation. Her condition improved with IV acyclovir, preventing the need for liver transplantation [7].

Furthermore, Timotijevic A et al. suggest that VZV infections are diagnostically challenging due to the absence of vesicular rash in approximately 5% of cases. Therefore, they recommend early recognition and timely administration of antiviral treatment in disseminated varicella-zoster virus infections, as it can be quickly fatal [8]. Toda T et al. reported a case where a patient developed a hyperacute liver failure immediately after a living-donor liver transplant due to VZV. This case proved to be fatal due to an atypical clinical presentation without cutaneous lesions, making it a diagnostic challenge and delaying acyclovir treatment [9].

DILI can occur as a result of intrinsic or idiosyncratic mechanisms with multiple drugs as triggering factors exhibiting hepatocellular, cholestatic or mixed damage to the liver. The main treatment for DILI is to discontinue the offending agent [10].

Among other causes of drug-induced liver injury, antimicrobials, especially beta-lactam antibiotics, have been identified among the top culprits, with amoxiclav as the most common hepatotoxic agent. DILI development in patients with amoxiclav use has been well established in patients with HLA phenotype; however, non-HLA genes have failed to recognize an association between DILI and amoxiclav [11]. Bariş Kuzu U. et al. also reported a case where a 24-year-old man developed autoimmune hepatitis fifteen days after taking amoxiclav for a tooth abscess [12].

A review done in 2022 revealed that autoimmune hepatitis mimics drug-induced autoimmune hepatitis histologically by having portal lymphocytic infiltration, plasma cells and eosinophils with no clear distinguishing features [13]. Waleed K Al-Hamoudi demonstrated that autoimmune hepatitis is characterized by inflammatory histological liver changes and the presence of autoantibodies in blood [2].

In summary, our case describes the possibility of herpes zoster reactivation secondary to COVID-19, which may have triggered autoimmune liver injury due to molecular mimicry. Although the patient received amoxiclav during hospitalization, which might have worsened the LFTs, the discontinuation of amoxiclav did not result in improvement of the patient's condition, which is the mainstay of treatment in DILI. We report the resolution of the patient's illness through acyclovir. Therefore, we highlight the importance of recognizing the atypical presentation of disseminated herpes zoster without skin lesions early to avoid fatality.

## Conclusions

Although there was a confounding factor of drug-induced liver injury as opposed to HZV infection based on liver biopsy results, comparable patterns of inflammation on liver biopsies in both conditions and improvement of the patient's clinical status with antiviral therapy make HZV autoimmune hepatitis more likely. Similar clinical presentation of herpes zoster-related autoimmune hepatitis and drug-induced liver injury, with the absence of a characteristic herpes zoster rash, makes the diagnosis of disseminated HZV infection challenging. To conclude, we recommend early evaluation for herpes zoster infection in any patient developing autoimmune hepatitis, especially in immunocompromise states, due to high mortality risk in disseminated infections. Prompt administration of antiviral medications can improve survival. Would also recommend further studies to identify the similar clinical presentation of DILI vs HZV-triggered autoimmune hepatitis.

## References

[1] Muratori L, Lohse AW, Lenzi M (2023). Diagnosis and management of autoimmune hepatitis. BMJ.

[2] Al-Hamoudi WK (2009). Severe autoimmune hepatitis triggered by varicella zoster infection. World J Gastroenterol.

[3] Chakravarty EF (2008). Viral infection and reactivation in autoimmune disease. Arthritis Rheum.

[4] Björnsson HK, Björnsson ES (2022). Drug-induced liver injury: Pathogenesis, epidemiology, clinical features, and practical management. Eur J Intern Med.

[5] Alharbi S, Alsubaie M, Alzayyat R, Alattas B, AlAhmadi H, Alabdullatif H (2023). Herpes zoster virus reactivation in a 16 year old female post COVID-19 vaccine. Case report and review of the literature. Med Arch.

[6] Czech T, Nishimura Y (2022). Characteristics of herpes zoster infection in patients with COVID-19: A systematic scoping review. Int J Dermatol.

[7] Brewer EC, Hunter L (2018). Acute liver failure due to disseminated varicella zoster infection. Case Reports Hepatol.

[8] Timotijevic A, Kodela P, Glušac V (2025). Disseminated varicella-zoster virus infection with internal organ involvement: A scoping review of 156 cases. Viruses.

[9] Toda T, Kaneko J, Ikemura M (2024). Fatal hyperacute liver failure due to varicella zoster virus immediately after living-donor liver transplantation: A case report and review of the literature. Pediatr Transplant.

[10] Hamilton LA, Collins-Yoder A, Collins RE (2016). Drug-induced liver injury. AACN Adv Crit Care.

[11] Alshabeeb MA, Aithal GP, Daly AK (2020). Investigation of oxidative stress-related candidate genes as risk factors for drug-induced liver injury due to co-amoxiclav. DNA Cell Biol.

[12] Kuzu UB, Oztaş E, Kaplan M, Suna N, Kekli̇k TT, Akdoğan M (2016). Drug induced autoimmune hepatitis by amoxicillin-clavulanate. Iran J Public Health.

[13] Tan CK, Ho D, Wang LM, Kumar R (2022). Drug-induced autoimmune hepatitis: A minireview. World J Gastroenterol.

